# Selective Modulation of Wnt Ligands and Their Receptors in Adipose Tissue by Chronic Hyperadiponectinemia

**DOI:** 10.1371/journal.pone.0067712

**Published:** 2013-07-04

**Authors:** Nobuhiko Wada, Toshihiko Hashinaga, Shuichi Otabe, Xiaohong Yuan, Yayoi Kurita, Satomi Kakino, Tsuyoshi Ohoki, Hitomi Nakayama, Tomoka Fukutani, Yuji Tajiri, Kentaro Yamada

**Affiliations:** Division of Endocrinology and Metabolism, Department of Medicine, Kurume University School of Medicine, Kurume, Fukuoka, Japan; National Cancer Center, Japan

## Abstract

**Background:**

Adiponectin-transgenic mice had many small adipocytes in both subcutaneous and visceral adipose tissues, and showed higher sensitivity to insulin, longer life span, and reduced chronic inflammation. We hypothesized that adiponectin regulates Wnt signaling in adipocytes and thereby modulates adipocyte proliferation and chronic inflammation in adipose tissue.

**Materials and Methods:**

We examined the expression of all Wnt ligands and their receptors and the activity of Wnt signaling pathways in visceral adipose tissue from wild-type mice and two lines of adiponectin-transgenic mice. The effects of adiponectin were also investigated in cultured 3T3-L1 cells.

**Results:**

The *Wnt5b*, *Wnt6*, Frizzled 6 (*Fzd6*), and *Fzd9* genes were up-regulated in both lines of transgenic mice, whereas *Wnt1*, *Wnt2*, *Wnt5a*, *Wnt9b*, *Wnt10b*, *Wnt11*, *Fzd1*, *Fzd2*, *Fzd4*, *Fzd7*, and the Fzd coreceptor low-density-lipoprotein receptor-related protein 6 (*Lrp6*) were reduced. There was no difference in total β-catenin levels in whole-cell extracts, non-phospho-β-catenin levels in nuclear extracts, or mRNA levels of β-catenin target genes, indicating that hyperadiponectinemia did not affect canonical Wnt signaling. In contrast, phosphorylated calcium/calmodulin-dependent kinase II (p-CaMKII) and phosphorylated Jun N-terminal kinase (p-JNK) were markedly reduced in adipose tissue from the transgenic mice. The adipose tissue of the transgenic mice consisted of many small cells and had increased expression of adiponectin, whereas cyclooxygenase-2 expression was reduced. *Wnt5b* expression was elevated in preadipocytes of the transgenic mice and decreased in diet-induced obese mice, suggesting a role in adipocyte differentiation. Some Wnt genes, Fzd genes, and p-CaMKII protein were down-regulated in 3T3-L1 cells cultured with a high concentration of adiponectin.

**Conclusion:**

Chronic hyperadiponectinemia selectively modulated the expression of Wnt ligands, Fzd receptors and LRP coreceptors accompanied by the inhibition of the Wnt/Ca^2+^ and JNK signaling pathways, which may be involved in the altered adipocyte cellularity, endogenous adiponectin production, and anti-inflammatory action induced by hyperadiponectinemia.

## Introduction

Visceral adipose tissue in metabolic syndrome is histologically characterized by enlargement of adipocytes due to impaired adipocyte differentiation, accompanied by chronic low-grade inflammation. The enlarged adipocytes release more free fatty acids, glycerol, and proinflammatory cytokines and less adiponectin. Hypoadiponectinemia and chronic inflammation in adipose tissue are closely associated with obesity-linked complications, including type 2 diabetes, coronary heart disease, and non-alcoholic fatty liver disease.

Previously, we established transgenic mouse lines that express full-length human adiponectin in the liver [1]. The hyperadiponectinemic mice show higher sensitivity to insulin, longer life span and resistance to the deleterious effects of a high-fat/high-sugar diet. The high-calorie diet-induced increase in urinary 8-hydroxy-2-deoxyguanosine, a marker of oxidative DNA damage, is markedly suppressed in the transgenic mice. Interestingly, adipocytes of the transgenic mice are reduced in size and increased in number compared with those of wild-type mice in both subcutaneous and visceral adipose tissues, suggesting that adiponectin may play a role in the regulation of adipogenesis. However, the mechanism of adiponectin action in adipose tissue has not been elucidated.

The Wnt signaling pathway is a highly conserved signal transduction cascade that has a critical role in embryonic development, differentiation and cellular homeostasis. Constitutive endogenous Wnt signaling keeps preadipocytes in an undifferentiated state [2]–[4]. However, Wnt signaling is involved in the activation of proinflammatory mediators in inflammatory disorders [5]–[7]. Wnt family members are involved in the regulation of many biological processes, including embryonic development, cell fate, cell proliferation, cell migration, stem cell maintenance, tumor suppression, and oncogenesis [8]. Binding of Wnt to the Frizzled (Fzd) family of receptors can activate at least two distinct signaling pathways. The canonical Wnt/β-catenin pathway is characterized by cytosolic and nuclear β-catenin accumulation and the activation of certain β-catenin-responsive target genes such as c-Myc (*Myc*) and cyclin D1 (*Ccnd1*) [9], [10]. The non-canonical β-catenin-independent pathways include the calcium/calmodulin-dependent kinase II (CaMKII)-mediated Wnt/Ca^2+^ pathway and the small GTPase RhoA- and Jun N-terminal kinase (JNK)-dependent planar cell polarity (PCP) pathway [11], [12]. In addition to Fzd receptors, the canonical Wnt/β-catenin signaling pathway requires low-density-lipoprotein receptor-related protein 5 (Lrp5) and Lrp6 coreceptors [13]–[15]. Furthermore, receptor tyrosine kinases-like orphan receptor 1 (Ror1), Ror2 and receptor-like tyrosine kinase (Ryk) transduce non-canonical Wnt signals and modulate Wnt/β-catenin signaling [16]–[19].

To test the hypothesis that adiponectin regulates Wnt signaling in adipocytes and thereby modulates adipocyte proliferation and chronic inflammation in adipose tissue, we examined the expression of all Wnt ligands and their receptors and the activities of both canonical and non-canonical Wnt signaling in visceral adipose tissue from wild-type mice and adiponectin-transgenic mice.

## Materials and Methods

### Animals

We have established three lines of transgenic mice expressing human full-length adiponectin under the control of the serum amyloid P component promoter on the C57BL/6 background [1]. In this study, we used male transgenic mice of line 11 and line 13, which exhibited a high plasma concentration of human adiponectin (284.1±44.4 and 183.0±32.0 µg/ml, respectively, including the high-molecular-weight isoform) [1]. The high plasma adiponectin enabled us to analyze adiponectin actions in adipose tissue, where adiponectin concentration is assumed to be higher than in peripheral blood. Wild-type C57BL/6 mice originally purchased from Nippon CLEA (Shizuoka, Japan) and maintained in our laboratory served as controls. Plasma adiponectin was 41.9±5.4 µg/ml in wild-type mice [1]. Mice were fed standard mouse chow (347 kcal/100 g, protein 24.9 g/100 g, fat 4.6 g/100 g; Nippon CLEA) and water ad libitum and sacrificed at the age of 20 weeks. To study diet-induced obesity, wild-type mice were maintained on a high-fat/high-sucrose food (592 kcal/100 g, 70% fat, 14% sucrose, 3% other carbohydrates, and 13% protein, Oriental Yeast, Tokyo, Japan) from the age of 8 weeks. This study was carried out in strict accordance with the recommendations in the Guide for the Care and Use of Laboratory Animals of the National Institutes of Health. All procedures were approved by the Ethics Review Committee for Animal Experimentation of Kurume University School of Medicine (Permit Number: 2262). Mice were sacrificed under ether anesthesia, and all efforts were made to minimize suffering.

### Quantitative Real-time RT-PCR

The mRNA expression of Wnt ligands, Wnt receptors, coreceptors, and components of Wnt signaling pathways was assessed by quantitative real-time RT-PCR. Visceral adipose tissue and quadriceps femoris muscle were obtained from 20-week-old male mice. Preadipocytes and mature adipocytes were isolated by the collagenase digestion method [20]. RNA was isolated using RNA-Bee (Cosmo Bio, Tokyo, Japan), and 5 µg of total RNA was reverse-transcribed to cDNA using a kit from Invitrogen (Carlsbad, CA, USA). SYBR green-based real-time quantitative PCR of cDNA templates was performed using StepOnePlus (Applied Biosystems, Foster City, CA, USA). The PCR cycling conditions were 10 min at 95°C followed by 40 cycles of 30 sec at 95°C, 30 sec at 53–64°C, and 30 sec at 72°C. The results were calculated as the expression of the target gene relative to the expression of the glyceraldehyde-3-phosphate dehydrogenase (*Gapdh*) gene. Forward and reverse primers are shown in Table 1. The expression of *Fabp4* and *Pparg* was measured by quantitative RT-PCR as markers of mature adipocytes.

**Table 1:** Primers for quantitative real-time RT-PCR.

Gene	Forward primer (5′ to 3′)	Reverse primer (5′ to 3′)	Amplicon (bp)
Wnt1	ctcatgaaccttcacaacaacga	atcccgtggcatttgca	80
Wnt2	ctccctctgctcttgacctg	ggcctggcacattgtcacac	106
Wnt2b	cgttcgtctatgctatctcgtcag	acaccgtaatggatgttgtcactac	169
Wnt4	aggatgctcggacaacatcgc	acgccagcacgtctttacctc	208
Wnt5a	agggaacgaatccacgctaagg	acaggctacatctgccaggttg	111
Wnt5b	agctgctgactgacgccaactc	gcgccaatgatgaacatctccg	79
Wnt6	tgcccgaggcgcaagactg	attgcaaacacgaaagctgtctctc	138
Wnt7b	tactacaaccaggcggaagg	gtggtccagcaagttttggt	233
Wnt8b	gtacaccctgactagaaactgc	ctgcttggaaattgcctctccg	145
Wnt9a	ggacaacctcaagtacagcag	tccactccagcctttatcacc	129
Wnt9b	acagcaccaagttcctcagc	cttgcaggttgttctcaggc	131
Wnt10	bgctgtaaccacgacatggacttc	ggcatttgcacttccgcttcagg	156
Wnt11	ctgaatcagacgcaacactgtaaac	ctctctccaggtcaagcaggtag	205
Fzd1	gcgacgtactgagcggagtg	tgatggtgcggatgcggaag	150
Fzd2	ctcaaggtgccgtcctatctcag	gcagcacaacaccgaccatg	156
Fzd3	ggtgtcccgtggcctgaag	acgtgcagaaaggaatagccaag	194
Fzd4	gacaactttcacgccgctcatc	ccaggcaaacccaaattctctcag	181
Fzd6	tgttggtatctctgcggtcttctg	ctcggcggctctcactgatg	110
Fzd7	atatcgcctacaaccagaccatcc	aaggaacggcacggaggaatg	194
Fzd8	gttcagtcatcaagcagcaaggag	aaggcaggcgacaacgacg	122
Fzd9	atgaagacgggaggcaccaatac	tagcagacaatgacgcaggtgg	107
Fzd10	atcggcacttccttcatcctgtc	tcttccagtagtccatgttgag	199
Ror1	ccccgatttcccaattacatg	gccaatgaaaccagcgatct	72
Ror2	tggaactgtgtgacgtaccc	gcgaggccatcagctg	186
Ryk	ccggctgcttggtcttgatgca	gtctcactgggcactagcaggtta	96
Lrp5	acccgctggacaagttcatc	tctgggctcaggctttgg	115
Lrp6	agggtggaatgagtgtgcct	tgatggcgctcttctgactga	171
Myc	gggccagccctgagcccctagtgc	atggagatgagcccgactccgacc	155
Ccnd1	ctggccatgaactacctgga	atccgcctctggcattttgg	279
Fabp4	ttggtcaccatccggtcaga	ttccaccaccagcttgtcac	207
Pparg	gtgaagcccatcgaggacat	acgtgctctgtgacgatctg	143

### Western Blot Analysis

Adipose tissue and 3T3-L1 cells were lysed in ice-cold lysis buffer containing 1 mmol/l dithiothreitol DTT, 0.0025% NP40 and a cocktail of proteinase inhibitors. The lysate was centrifuged at 19,000 *g* for 15 min at 4°C, and the supernatant was collected as whole-cell extract. To obtain nuclear extract, adipose tissue lysate was centrifuged at 600 *g* for 15 min at 4°C, and the pellet was solubilized in nuclear lysis buffer containing 0.5 mmol/l DTT, 20% glycerol, 0.2 mmol/l EDTA and protease inhibitors. After centrifugation at 12,000 *g* for 10 min, the supernatant was collected. The total protein concentrations of the whole-cell and nuclear extracts were measured using the Bradford reagent (Bio Rad, Hercules, CA, USA). After being heated at 100°C for 5 min, 20 µg total protein was loaded into each well, separated by 7.5% SDS-PAGE (Wako, Osaka, Japan) and transferred to a nitrocellulose membrane. The membrane was incubated with rabbit polyclonal antibodies against β-catenin and non-phospho β-catenin, rabbit polyclonal antibody against CaMKII, rabbit polyclonal antibody against phospho-CaMKII Thr286 (p-CaMKII), rabbit monoclonal antibody against JNK, or rabbit monoclonal antibody against phospho-JNK Thr183/Thr185 (Cell Signaling Technology, Danvers, MA, USA) at 4°C overnight. After being washed, the membrane was incubated with peroxidase-conjugated goat anti-rabbit IgG (Wako) and then visualized using an ECL system (GE Healthcare, Buckinghamshire, UK).

### 3T3-L1 Cell Culture

3T3-L1 cells were purchased from DS Pharma Biomedical (Osaka, Japan) and used when the cells were nearly confluent. The cells were cultured with 50, 150, or 300 µg/ml of recombinant human adiponectin (Biovendor, Candler, NC, USA) for 24 h. Thereafter, RNA was isolated from the cells, and the mRNA levels of Wnt-related genes were assessed by quantitative real-time RT-PCR as described above. To assess the effect of adiponectin on the non-canonical Wnt signaling pathways, 3T3-L1 cells were cultured with 50, 150, or 300 µg/ml of recombinant human adiponectin, 1 µg/ml of recombinant human Wnt5a (R&D Systems, Minneapolis, MN, USA) or both for 24 h. Wnt5A was shown to be biologically active at the concentration [21], [22]. Total and phosphorylated JNK and CaMKII were analyzed by Western blotting.

### Statistical Analysis

Quantitative results are expressed as means and SD. Statistical analysis was performed using the unpaired Student’s *t* test for comparison between two groups after confirming the normality of the data distribution. *P* values less than 0.05 were considered statistically significant.

## Results

### Expression of Wnt Ligands

**Figure 1:**
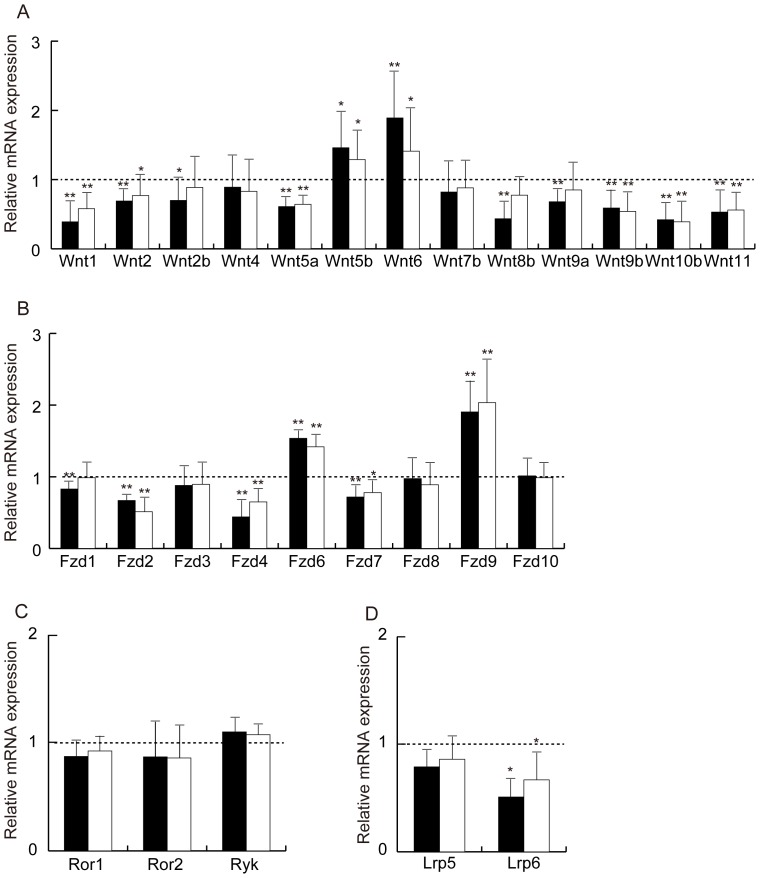
Analysis of the Wnt ligand family and their receptors in adipose tissue from wild-type mice and adiponectin-transgenic mice. mRNA levels of the Wnt ligand family (A), Frizzled receptors (B), non-Fzd Wnt receptors (C) and coreceptors (D) in adipose tissue from line 11 (closed bars) and line 13 (open bars) of male adiponectin-transgenic mice. Data are shown as ratios to wild-type C57BL/6 mice at the age of 20 weeks (n = 6–10, for each group). mRNA levels were determined by quantitative real-time RT-PCR and normalized to *Gapdh* mRNA. Means and SD, *p<0.05, **p<0.01 vs. wild-type mice.

Quantitative RT-PCR analyses revealed that numerous Wnt ligands and their receptors were expressed in visceral adipose tissue. Of the 19 known mouse *Wnt* genes, 13 were detectable: *Wnt1*, *Wnt2*, *Wnt2b*, *Wnt4*, *Wnt5a*, *Wnt5b*, *Wnt6*, *Wnt7b*, *Wnt8b*, *Wnt9a*, *Wnt9b*, *Wnt10b* and *Wnt11* (Fig. 1A). Adiponectin-transgenic mice of line 11 and line 13 showed up-regulated expression of *Wnt5b* and *Wnt6* compared with wild-type mice, whereas *Wnt1*, *Wnt2*, *Wnt5a*, *Wnt9b*, *Wnt10b*, and *Wnt11* were significantly reduced in both transgenic lines. In addition, the expression of *Wnt2b*, *Wnt8b*, and *Wnt9a* was decreased in line 11 transgenic mice, which had higher circulating adiponectin than line 13.

### Expression of Wnt Receptors and Coreceptors

Next, we analyzed the expression of Wnt receptor genes. Nine out of 10 known *Fzd* genes were expressed in adipose tissue (Fig. 1B). The expression of *Fzd6* and *Fzd9* was significantly increased in both lines of adiponectin-transgenic mice, while that of *Fzd1*, *Fzd2*, *Fzd4* and *Fzd7* was reduced compared with wild-type mice. However, the expression of *Ror1*, *Ror2* and *Ryk* was not different between the transgenic mice and wild-type mice (Fig. 1C). We further examined the expression of *Lrp5* and *Lrp6* coreceptor genes and found that *Lrp6* mRNA was markedly decreased in adipose tissue from the transgenic mice (Fig. 1D).

### Analysis of Canonical Wnt Signaling Pathway

**Figure 2:**
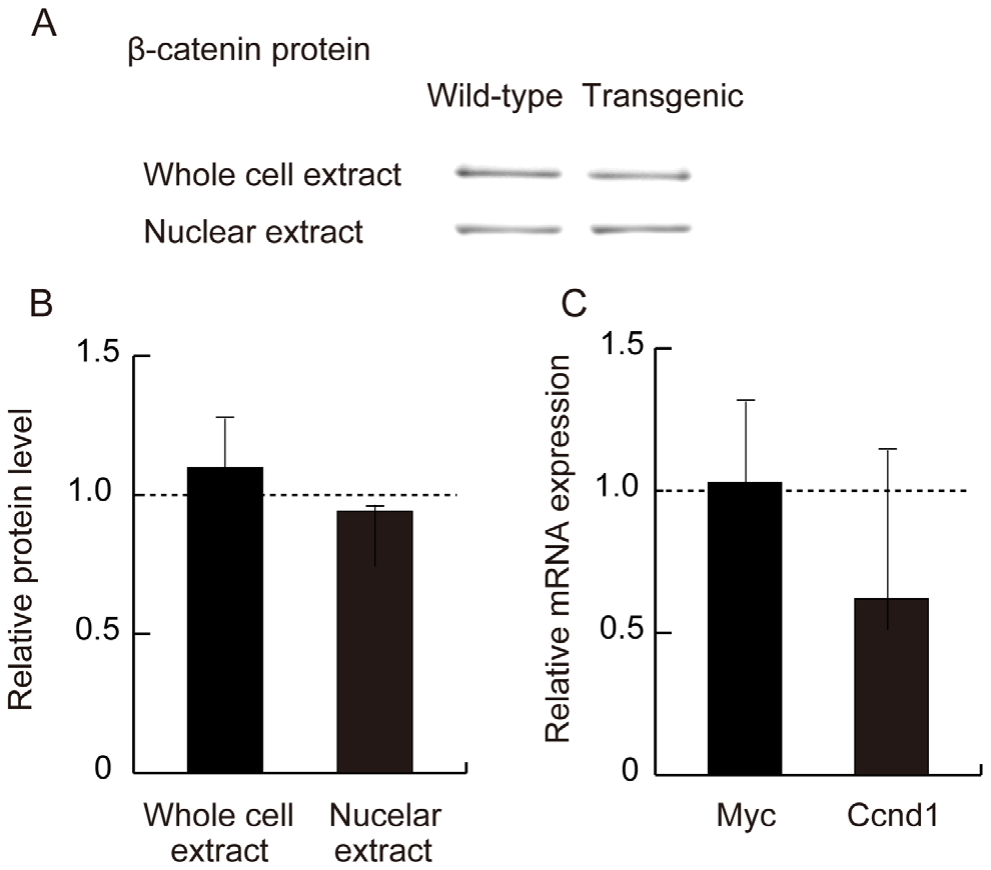
Analysis of the canonical Wnt signaling pathway in adipose tissue from wild-type mice and adiponectin-transgenic mice at the age of 20 weeks. (A) Western blot analysis of total β-catenin in whole-cell extracts and non-phosphorylated (active form) β-catenin in nuclear extracts. (B) Relative protein levels of total β-catenin in whole-cell extracts and non-phospho-β-catenin in nuclear extracts of adipose tissue from transgenic mice shown as ratios to those of wild-type mice (n = 5, each). (C) mRNA expression of c-Myc (*Myc*) and cyclin D1 (*Ccnd1*) genes in transgenic mice shown as ratios to wild-type mice (n = 10, each). Means and SD.

To assess canonical Wnt signaling in adipose tissue, β-catenin was measured by Western blot analysis. There was no difference in total β-catenin levels in whole-cell extracts or in non-phospho-β-catenin levels in nuclear extracts between wild-type mice and the transgenic mice (Fig. 2A, B). Furthermore, wild-type and transgenic mice showed equivalent mRNA levels of β-catenin target genes c-Myc (*Myc*) and cyclin D1 (*Ccnd1*) (Fig. 2C).

### Analysis of Non-canonical Wnt Signaling Pathways

**Figure 3:**
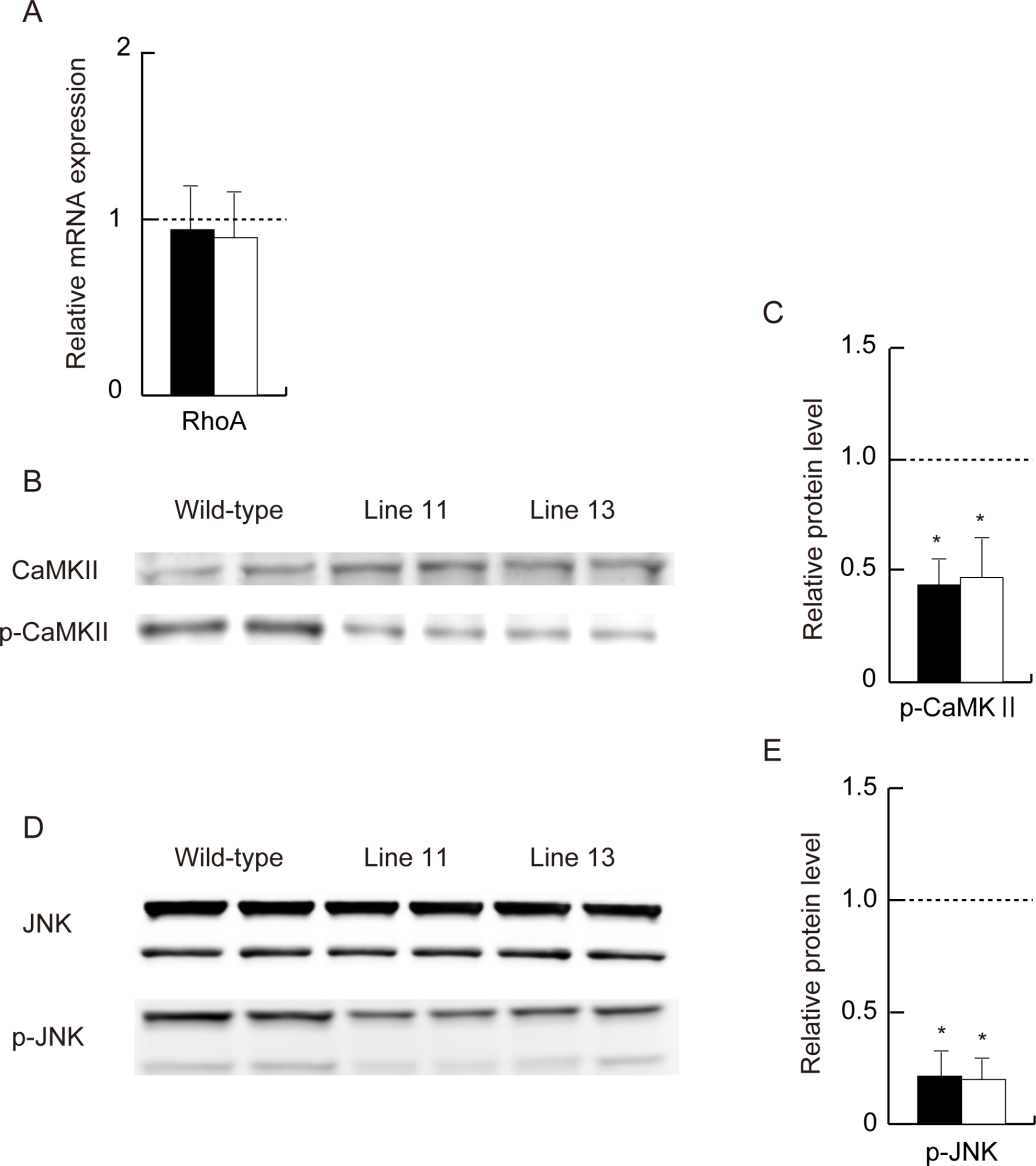
Analysis of the non-canonical Wnt signaling pathway in adipose tissue from wild-type mice and line 11 (closed bars) and line 13 (open bars) of transgenic mice at the age of 20 weeks. (A) Expression of *RhoA* in adiponectin-transgenic mice shown as a ratio to that in wild-type mice (n = 6–10, for each group). Western blot analysis of total and phosphorylated CaMKII (B) and the relative protein levels of p-CaMKII (C) in transgenic mice compared with wild-type mice (n = 5, each). Western blot analysis of total and phosphorylated JNK (D) and the relative protein levels of p-JNK (E) in transgenic mice compared with wild-type mice (n = 4, each). Means and SD, *p<0.01 vs. wild-type mice.

The expression of *RhoA* in adipose tissue was not different between the transgenic mice and wild-type mice (Fig. 3A). The protein level of CaMKII, another non-β-catenin-dependent Wnt pathway signaling molecule, was not altered in either line of transgenic mice. However, p-CaMKII, the active form of CaMKII, was significantly reduced in adipose tissue from the transgenic mice (Fig. 3B, C). Similarly, p-JNK was markedly down-regulated in both lines of transgenic mice, while total JNK was comparable between the transgenic mice and wild-type mice (Fig. 3D, E).

### Expression of Endogenous Adiponectin and Cyclooxygenase-2

**Figure 4:**
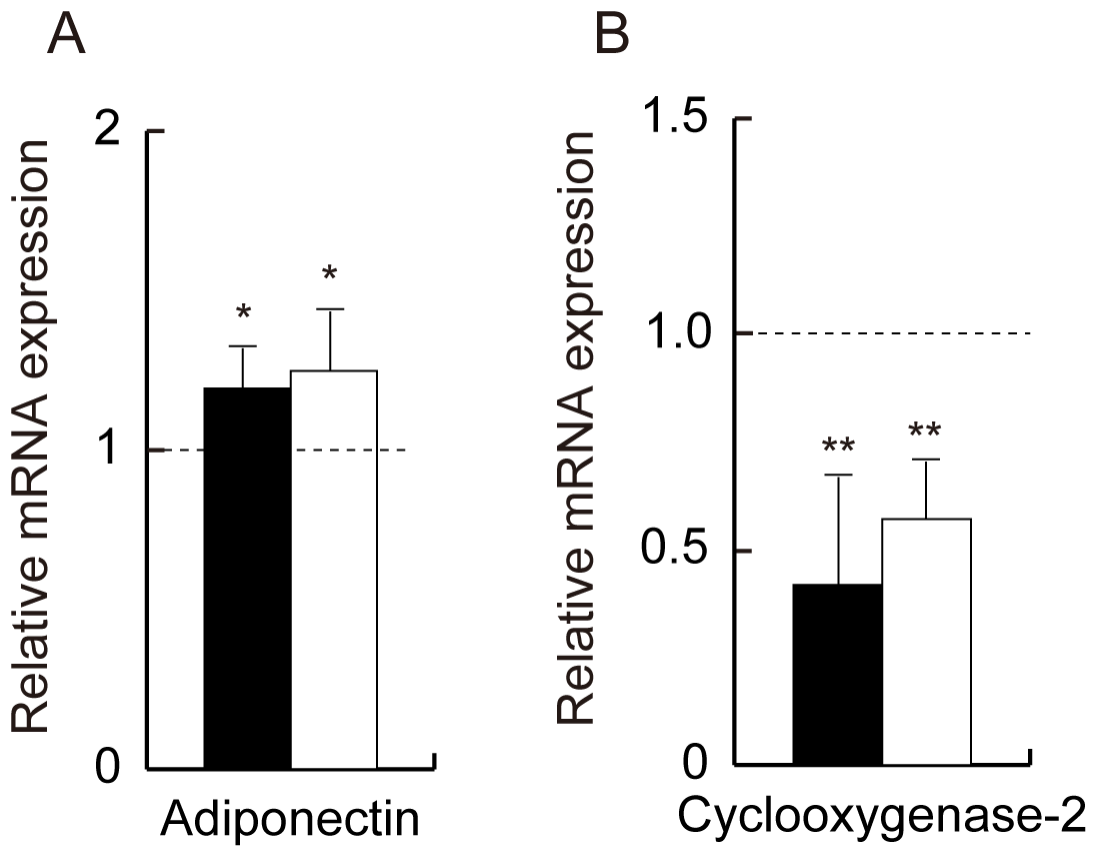
mRNA levels of the mouse adiponectin and cyclooxygenase-2 in adipose tissue from wild-type mice and adiponectin-transgenic mice. Expression of mouse adiponectin (A) and cyclooxygenase-2 (B) in adipose tissue from line 11 (closed bars) and line 13 (open bars) of adiponectin-transgenic mice shown as ratios to those of wild-type mice at the age of 20 weeks (n = 6–10, each). Relative mRNA expression was normalized to *Gapdh* expression. Means and SD, *p<0.05, **p<0.01 vs. wild-type mice.

The expression of the endogenous mouse adiponectin gene was increased in the adiponectin-transgenic mice compared with the wild-type mice (Fig. 4A). The hyperadiponectinemic mice of both lines exhibited significantly lower cyclooxygenase-2 mRNA in adipose tissue (Fig. 4B).

### Association of Wnt Ligand Gene Expression with Adipocyte Differentiation and Diet-induced Obesity

**Figure 5:**
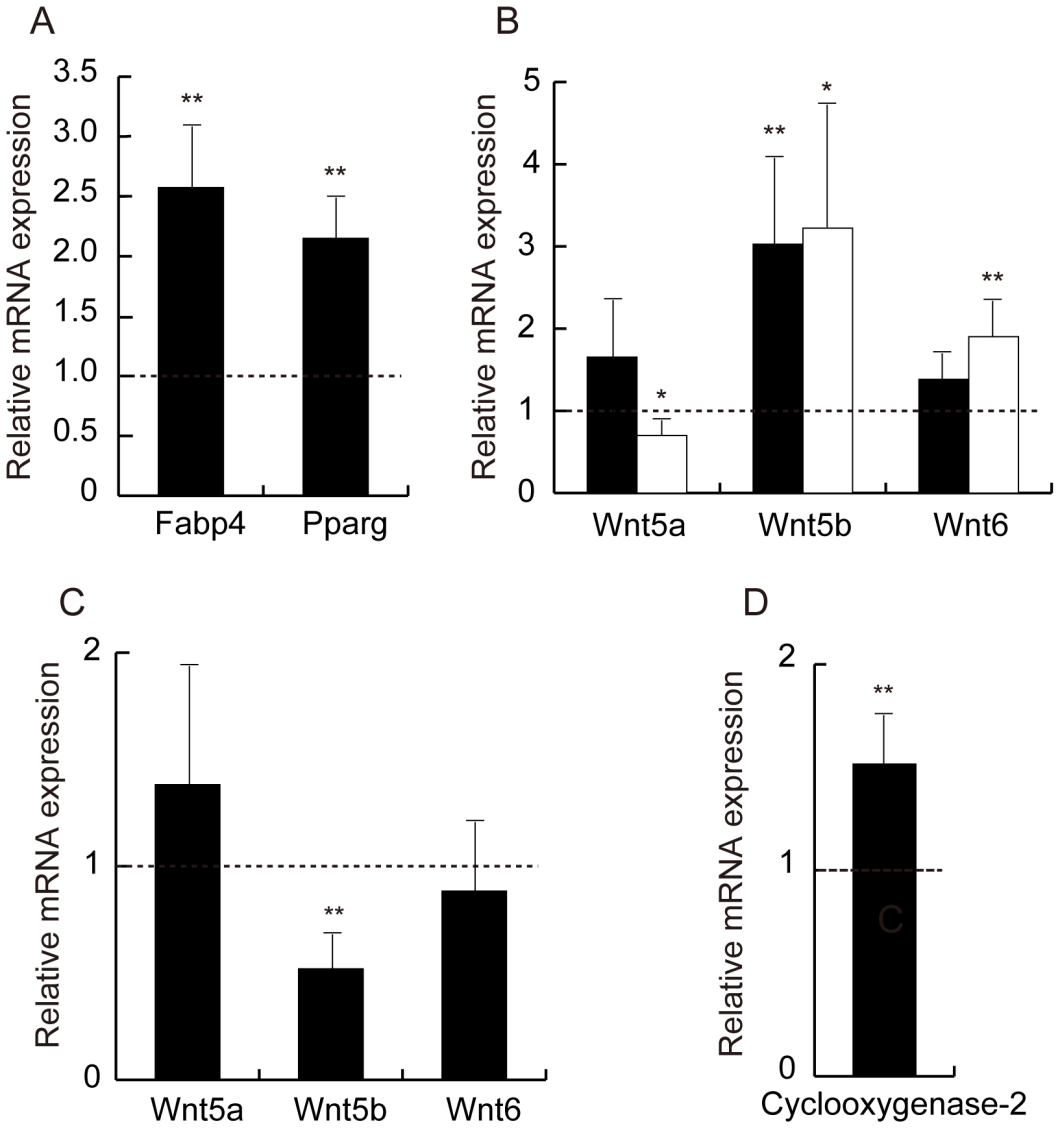
Analysis of the preadipocyte fractions and mature adipocyte fractions in adipose tissue from wild-type mice and adiponectin-transgenic mice. mRNA levels of the Wnt ligands (A) in preadipocyte fractions (closed bars) and mature adipocyte fractions (open bars) from adiponectin-transgenic mice shown as ratios to those of wild-type C57BL/6 mice at the age of 20 weeks (n = 4 or 5, for each group). mRNA levels of adipocyte marker genes in the mature adipocyte fractions shown as ratios to those of the preadipocyte fractions (B). Expression of Wnt ligands (C) and the cyclooxygenase-2 gene (D) in adipose tissue from diet-induced obese mice shown as ratios to those in wild-type mice (n = 4, each). Means and SD. *p<0.05, **p<0.01 vs. wild-type mice.

The expression of adipocyte marker genes, *Fabp4* and *Pparg*, was higher in the mature adipocyte fraction than in the preadipocyte fraction confirming that two cell populations were isolated (Fig. 5A). The elevation of *Wnt5b* expression was observed in both preadipocyte and mature adipocyte fractions from adiponectin-transgenic mice. However, the reduction of *Wnt5a* mRNA and the increase of *Wnt6* mRNA were observed only in the mature adipocyte fraction (Fig. 5B). The expression of *Wnt5b* was significantly reduced in adipose tissue from obese mice maintained on the high-fat/high-sucrose food for 12 weeks (Fig. 5C) compared with wild-type mice fed normal mouse chow (body weight 47.8±0.9 vs. 32.4±2.0 g, respectively, n = 4 each, p<0.0001). Cyclooxygenase-2 was elevated in the adipose tissue of the mice fed the high-energy food (Fig. 5D).

### Effects of Adiponectin on Wnt Signaling Components in 3T3-L1 Cells

**Figure 6:**
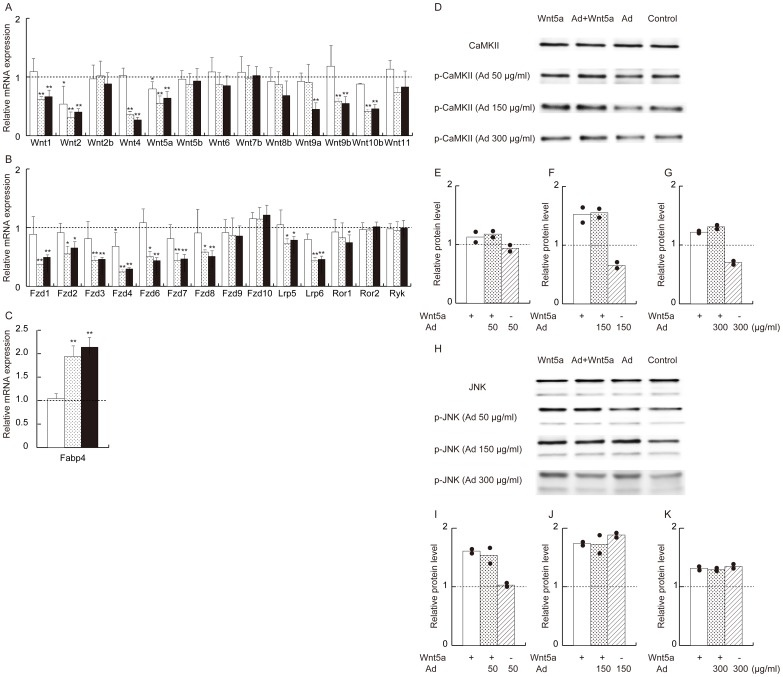
Effects of adiponectin on Wnt signaling components of 3T3-L1 cells. mRNA levels of the Wnt ligand family (A), Wnt receptors (B), and Fabp4 (C) in 3T3-L1 cells cultured with 50 (open bars), 150 (dotted bars), and 300 µg/ml (closed bars) of adiponectin shown as ratios to those of 3T3-L1 cells without adiponectin supplementation (n = 3, each). Means and SD. *p<0.05, **p<0.01. Protein levels of total and phosphorylated forms of CaMKII (D) and JNK (H) and relative protein levels of p-CaMKII (E, F, G) and p-JNK (I, J, K) in 3T3-L1 cells incubated with Wnt5a (open bars), Wnt5a and 50 (E, I), 150 (F, J), or 300 µg/ml (G, K) of adiponectin (dotted bars), or 50 (E, I), 150 (F, J), or 300 µg/ml (G, K) of adiponectin alone (hatched bars) compared with non-treated 3T3-L1 cells. The dose-response experiments were carried out independently at each concentration. Western blot analyses were performed twice with essentially the same results. Dots indicate individual data points (E, F, G, I, J, K). Ad, adiponectin.

The exposure of 3T3-L1 cells to 300 µg/ml of adiponectin for 24 h resulted in the reduction of *Wnt1*, *Wnt2*, *Wnt4*, *Wnt5a*, *Wnt9a*, *Wnt9b*, and *Wnt10b* expression (Fig. 6A). The mRNA levels of *Fzd1*, *Fzd2*, *Fzd3*, *Fzd4*, *Fzd6*, *Fzd7*, *Fzd8*, *Lrp5*, *Lrp6*, and *Ror1* were also decreased after culture in the presence of 300 µg/ml of adiponectin (Fig. 6B). Adiponectin of 150 µg/ml down-regulated the expression of these genes except *Wnt9a* and *Ror1*. The expression of *Fabp4*, a marker of adipocyte maturation, was increased in 3T3-L1 cells cultured with 150 or 300 µg/ml of adiponectin (Fig. 6C). Western blot analysis showed that 24-h incubation of 3T3-L1 cells with adiponectin and/or Wnt5a did not alter the intracellular protein level of total CaMKII or total JNK (Fig. 6D, H). However, p-CaMKII was reduced in 3T3-L1 cells treated with 150 or 300 µg/ml adiponectin but increased in Wnt5a-treated cells compared with non-treated cells. Supplementation with adiponectin did not inhibit the Wnt5a-induced elevation of p-CaMKII (Fig. 6D, E, F, G). In contrast, p-JNK increased not only in Wnt5a-treated cells but also in cells treated with 150 or 300 µg/ml adiponectin (Fig. 6H,I,J,K).

### Expression of Wnt Ligand and Wnt Receptor Genes in Skeletal Muscle

**Figure 7:**
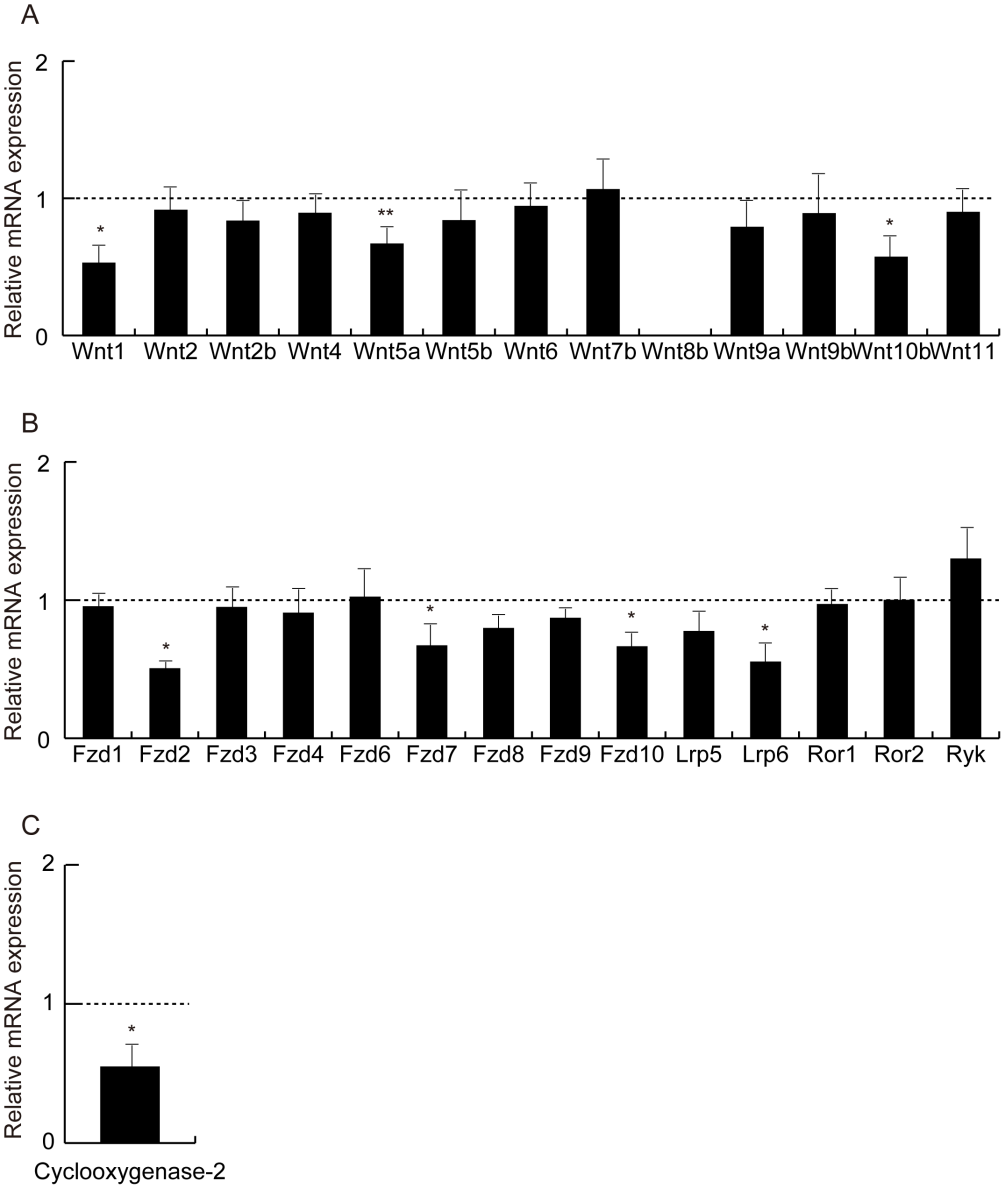
Analysis of the Wnt ligand family, their receptors and cyclooxygenase-2 in skeletal muscle from wild-type mice and adiponectin-transgenic mice. mRNA levels of the Wnt ligand family (A), Frizzled receptors (B), and the cyclooxygenase-2 gene (C) in skeletal muscle from adiponectin-transgenic mice shown as ratios to those of wild-type C57BL/6 mice at the age of 20 weeks (n = 4, each). Means and SD. *p<0.05, **p<0.01 vs. wild-type mice.

The effects of hyperadiponectinemia on Wnt-related gene expression in skeletal muscle were examined for comparison with those in adipose tissue. The mRNA levels of *Wnt1*, *Wnt5a*, *Wnt10b*, *Fzd7*, *Fzd10*, and *Lrp6* were significantly lower in the skeletal muscle from adiponectin transgenic mice (line 11) than wild-type mice (Fig. 7A, B). The expression of cyclooxygenase-2 was reduced in the skeletal muscle from the transgenic mice (Fig. 7C).

## Discussion

In this comprehensive analysis of Wnt ligands and their receptors in adipose tissue, we demonstrated that hyperadiponectinemia elicited profound effects on Wnt pathway components. Of 13 detectable Wnt ligand families, both lines of adiponectin-transgenic mice showed increased expression of *Wnt5b* and *Wnt6* and reduced expression of *Wnt1*, *Wnt2*, *Wnt5a*, *Wnt9b*, *Wnt10b*, and *Wnt11*. Several Wnt ligands, including Wnt1, predominantly use the canonical pathway for intracellular signaling, while some other Wnt ligands, including Wnt5a, mainly activate non-canonical signaling [23], [24]. However, a growing body of evidence suggests substantial overlap between these pathways in cell- and context-dependent manners [25], [26].

Wnt ligands can signal through different receptors, including Fzd, Ror1, Ror2 and Ryk. Fzd receptors are seven-pass transmembrane proteins requisite for canonical Wnt signaling. Two members of the Fzd family were up-regulated and four were down-regulated in adipose tissue from the transgenic mice. However, no significant difference was observed in the expression of the non-Fzd Wnt receptors *Ror1*, *Ror2* and *Ryk* between wild-type mice and the transgenic mice. On the other hand, the hyperadiponectinemic mice showed lower expression of coreceptor *Lrp6*, whereas *Lrp5* expression was not significantly altered.

These observations prompted us to analyze the effects of hyperadiponectinemia on Wnt signaling pathways in adipose tissue. To assess the activity of the canonical signaling pathway, we examined β-catenin. Western blot analysis showed neither total β-catenin in whole-cell extracts nor non-phospho-β-catenin in nuclear extracts was altered in the transgenic mice compared to wild-type mice. Furthermore, no significant difference was observed in the expression of *Myc* or *Ccnd1*, classical target genes of the canonical Wnt signaling pathway [27]–[29], between wild-type mice and adiponectin-transgenic mice. Hence, chronic hyperadiponectinemia in the transgenic mice did not alter the downstream signals of the canonical Wnt pathway in adipose tissue. *Lrp5* expression might be sufficient to transduce canonical signals in spite of the reduction of *Lrp6* expression.

The two non-canonical Wnt signaling pathways are the Wnt/PCP pathway, involving RhoA GTPase and JNK, and the Wnt/Ca^2+^ pathway, involving CaMKII and protein kinase C. In this study the expression of *RhoA* was not altered in adipose tissue from the transgenic mice, although RhoA activity was not measured. However, the protein levels of p-JNK and p-CaMKII, but not total JNK and total CaMKII, were markedly reduced in the transgenic mice compared with wild-type mice. Although the physiological roles of individual Fzd members remain to be characterized, Wnt5a binds to Fzd2 and Fzd4 and transduces the Wnt/Ca^2+^ signal. The attenuated Wnt/Ca^2+^ signaling pathway in the transgenic mice could be attributable to the reduced expression of *Wnt5a* and its putative receptors *Fzd2* and *Fzd4*.

Insulin resistance and type 2 diabetes are associated with impaired adipogenesis, characterized by increased fat cell size. Previously, we reported that adipocytes are small in size but increase in number in adiponectin-transgenic mice fed a high-fat/high-sugar diet compared with wild-type mice fed the same diet [1]. The elevated expression of endogenous adiponectin shown in this study may have resulted from the increase of small adipocytes in the transgenic mice. Wnt signaling may play a role in the altered cellularity of hyperadiponectinemic mice because non-canonical Wnt signaling is one of the regulatory mechanisms of osteoblast and adipocyte differentiation from common mesenchymal stem cells [30], [31]. Wnt5a can shift the balance towards osteoblastogenesis through the activation of CaMKII [30]–[32]. Thus, the reduced expression of Wnt5a and lower level of p-CaMKII in the transgenic mice may have enhanced adipogenesis.

Unlike *Wnt5a*, adipose expression of *Wnt5b* and *Wnt6* was elevated in the transgenic mice. Overexpression of *Wnt5b* in 3T3-L1 preadipocytes results in the augmentation of adipocyte differentiation, suggesting that Wnt5b is a positive regulator of adipogenesis [33]. Wnt6 may also be involved in the regulation of adipogenesis because *Wnt6* mRNA shows biphasic expression during the differentiation period of 3T3-L1 cells [34]. Taken together, our data suggest that the altered expression of Wnt ligands in adipose tissue from the transgenic mice may be involved in the enhanced adipogenesis and thereby the up-regulated production of endogenous adiponectin.

Next, we examined the expression of putative regulators of adipocyte differentiation, Wnt5a, Wnt5b, and Wnt6, in preadipocytes and mature adipocytes. The expression of *Wnt5b* was elevated in both preadipocytes and mature adipocytes from adiponectin-transgenic mice, while the reduction of *Wnt5a* mRNA and the increase of *Wnt6* mRNA were detected only in mature adipocytes. These observations suggest that Wnt5b may play a major role in the adipocyte differentiation induced by hyperadiponectinemia. This suggestion is supported by the fact that diet-induced obesity resulted in the reduction of *Wnt5b* expression in adipose tissue. However, it should be noted that the preadipocyte fraction may also contain immune cells and endothelial cells.

Activated Wnt5a signaling has been implicated in the up-regulation of proinflammatory cytokines in autoimmune and infectious diseases [35], [36]. Serum levels of Wnt5a were increased in patients with severe obesity [37]. Wnt5a induces cyclooxygenase-2 expression by endothelial cells through the non-canonical Wnt/Ca^2+^ signaling pathway, leading to the enhancement of inflammatory cytokine production [7]. As expected, cyclooxygenase-2 expression was significantly reduced in adipose tissue from adiponectin-transgenic mice. Hence, the inhibition of Wnt/Ca^2+^ signaling may be a mechanism by which adiponectin attenuates the chronic inflammation associated with obesity and type 2 diabetes.

The promoter region of the *Wnt5a* gene contains a binding element of NF-κB, a transcription factor activated by TNF-α [38]. Down-regulation of NF-κB may mediate the suppressive effect of adiponectin on *Wnt5a* expression because adiponectin inhibits TNF-α-induced IκB-α phosphorylation and subsequent NF-κB activation [39], [40]. Further studies are needed to define the mechanism of the selective modulation of Wnt ligands and their receptors by adiponectin.

It was of interest that p-JNK was markedly reduced in adipose tissue from the transgenic mice because JNK has been implicated in the development of insulin resistance and diabetes. In obesity, JNK activity is increased in the liver, muscle, and adipose tissues, and an absence of JNK1 results in decreased adiposity and improved insulin sensitivity in obese models of mice [41]. Furthermore, JNK is a crucial regulator of apoptosis and cell proliferation. Activation of JNK might be associated with increased carcinogenesis in obesity [42], and it was hypothesized that decreased adiponectin levels, as observed in obesity, fail to control JNK expression. Although various inflammatory and metabolic pathways may be involved in the activation of JNK, the observations in this study suggest that the attenuation of non-canonical Wnt signaling is a mechanism by which hyperadiponectinemia suppresses JNK activity in adipose tissue.

Next, we assessed the *ex vivo* effects of adiponectin on the mRNA levels of Wnt ligands and their receptors using cultured 3T3-L1 cells. The cells were exposed to a high concentration of recombinant adiponectin (300 µg/ml) because adiponectin concentration is likely to be elevated in the micro-environment of adipocytes *in vivo*. As in adipose tissue from the transgenic mice, down-regulation of *Wnt1*, *Wnt2*, *Wnt5a*, *Wnt9b*, *Wnt10b*, *Fzd2*, *Fzd4*, *Fzd7*, and *Lrp6* was also detected in 3T3-L1 cells exposed to adiponectin for 24 h. Thus, adiponectin may directly attenuate the expression of these Wnt-related genes in adipose tissue. In contrast, the reduction of *Wnt11* expression in adipose tissue may be an indirect effect of hyperadiponectinemia because *Wnt11* mRNA was not decreased in 3T3-L1 cells cultured with adiponectin. The expression of some Wnt-related genes was reduced by adiponectin *in vitro*, but not in adipose tissue from the transgenic mice. Furthermore, up-regulation of *Wnt5b*, *Wnt6*, *Fzd6* and *Fzd9*, observed in the adipose tissue from the transgenic mice, was not observed in 3T3-L1 cells exposed to adiponectin. Although these discrepancies might be explained by the distinct responsiveness of 3T3-L1 cells or the relatively short-term of exposure to adiponectin in the culture experiment, the expression of some Wnt-related genes in adipose tissue of the transgenic mice might be modified by endocrinologic and metabolic alterations resulting from hyperadiponectinemia.

In accordance with the *in vivo* observations of adiponectin-transgenic mice, p-CaMKII was decreased in 3T3-L1 cells after 24-h culture with adiponectin. By contrast, p-CaMKII was elevated after exposure to Wnt5a. The supplementation with adiponectin failed to inhibit the activation of CaMKII induced by Wnt5a, suggesting that the suppressive effect of adiponectin on the non-canonical signaling pathway is mainly due to the modulation of Wnt ligand expression rather than alterations in their receptors. As expected, phosphorylation of JNK, another non-canonical signaling molecule, was augmented in cells incubated with Wnt5a. However, exposure of 3T3-L1 cells to adiponectin also resulted in activation of JNK. This finding is not surprising because it was reported that adiponectin stimulates JNK *ex vivo* through the activation of phospholipase C [43], [44]. The suppression of JNK activity observed in adiponectin-transgenic mice may have been a more chronic effect of adiponectin.

Finally, we analyzed the alterations of Wnt-related gene expression in skeletal muscle from the transgenic mice and found that the expression of *Wnt1*, *Wnt5a*, *Wnt10b*, *Fzd7*, *Fzd10*, and *Lrp6*, as well as the cyclooxygenase-2 gene, was reduced. All of the genes except *Fzd10* were also down-regulated in adipose tissue from the transgenic mice, although there was considerable organ-specificity in the response of the Wnt system to hyperadiponectinemia. The physiological relevance of the changes in Wnt-related gene expression in skeletal muscle found in this study requires further investigation.

In conclusion, using adiponectin-transgenic mice, we showed that chronic hyperadiponectinemia altered the expression pattern of Wnt ligands, Fzd receptors and the Lrp6 coreceptor in adipose tissue, suggesting that the inhibition of the Wnt/Ca^2+^ and JNK signaling pathways may be involved in the enhanced adipocyte differentiation and attenuated inflammation in adipose tissue induced by hyperadiponectinemia. Thus, adiponectin might exert these effects when adiponectin production is augmented by body weight loss or the administration of thiazolidine derivatives. However, further studies are required to elucidate the dose-response relationship and to determine whether adiponectin exhibits similar action in other tissues.
